# Improving sepsis care in Africa: an opportunity for change?

**DOI:** 10.11604/pamj.2021.40.204.30127

**Published:** 2021-12-06

**Authors:** Alexander James Keeley, Emmanuel Nsutebu

**Affiliations:** 1 Florey Institute, Department of Infection, Immunity and Cardiovascular Disease, University of Sheffield, Sheffield, United Kingdom,; 2 Infectious Disease Division, Sheikh Shakhbout Medical City, Abu Dhabi, United Arab Emirates

**Keywords:** Africa, sepsis, septic shock, surviving sepsis campaign, African sepsis alliance, antibiotics, intravenous fluids

## Abstract

Sepsis is common and represents a major public health burden with significant associated morbidity and mortality. However, despite substantial advances in sepsis recognition and management in well-resourced health systems, there remains a distinct lack of research into sepsis in Africa. The lack of evidence affects all levels of healthcare delivery from individual patient management to strategic planning at health-system level. This is particular pertinent as African countries experience some of the highest global burden of sepsis. The 2017 World Health Assembly resolution on sepsis and the creation of the Africa Sepsis Alliance provided an opportunity for change. However, progress so far has been frustratingly slow. The recurrent Ebola virus disease outbreaks and the COVID-19 pandemic on the African continent further reinforce the need for urgent healthcare system strengthening. We recommend that African countries develop national action plans for sepsis which should address the needs of all critically ill patients.

## Essay

### Introduction

Sepsis is a syndrome defined as life threatening organ dysfunction caused by a dysregulated host response to infection [1]. However despite a rising profile globally, there are many unanswered questions about sepsis in low and middle income countries [2]. The World Health Assembly (WHA) passed a resolution in May 2017, making sepsis a global health priority. This in turn has led to the creation of the African Sepsis Alliance to coordinate implementation of the resolution. The aim of this paper is to describe recent advances in understanding of sepsis management in Africa, highlight areas for further research and identify strategic goals to improve sepsis care in Africa. This paper also highlights the challenges faced by those tasked with enacting the WHA resolution.

### Why is this an important problem?

Sepsis represents a major global public health problem. Almost fifty million people worldwide develop sepsis every year, resulting in eleven million deaths annually. About 85% of cases occur in low and middle income countries such as countries in Africa. In addition, children (especially new borns), pregnant women and the elderly are the most affected. Most of these cases and deaths occur in Africa, resulting in an estimated 17 million cases and 3.5 million deaths in 2017 [3]. In addition, since sepsis affects younger people in Africa, it is associated with a disproportionate number of years of life lost. Sepsis is recognised as the most important preventable cause of death worldwide [3]. There is extensive research into strategies to improve survival in well-resourced healthcare systems. In addition, the Surviving Sepsis Campaign has successfully raised the profile of sepsis since its inception by raising awareness, providing evidence based guidelines for optimal management, education and performance improvement. Concurrently outcomes from sepsis have improved in well-resourced systems [4-6]. However, there is a lack of published research on sepsis management in resource-limited health systems [7]. In Africa where there is a higher burden of infectious disease including HIV, where resource limitations are greater, and where intensive care facilities are often not available, there is an urgent need for high quality research describing the burden of sepsis and evaluating interventions which improve survival. While there is some evidence that surviving sepsis guidance can be adapted for low resource settings, caution must be applied with direct implementation of guidance, particularly relating to intravenous fluid management [8-10]. Nonetheless, the mainstay of effective intervention in sepsis relies on prompt recognition, timely delivery of simple basic interventions such as administration of antibiotics and close monitoring of the response to treatment.

### How should sepsis be defined in Africa?

In February 2016 the 3^rd^ International Consensus definitions for sepsis and septic shock (Sepsis-3) were published [1]. Sepsis is now defined as “life threatening organ dysfunction caused by dysregulated host response to infection” as determined by a change in the sequential sepsis related organ failure score (SOFA score) of 2 or more (Table 1); validated retrospectively in large cohorts in USA and Germany. Septic shock is characterised by vasopressor requirement to maintain a mean arterial pressure of 65 mmHg or higher, and a serum lactate level greater than 2 mmol/L (> 18 mg/dL) in the absence of hypovolemia [1]. However, use of the SOFA score requires laboratory investigations and critical care measurements of levels of organ dysfunction that are not readily available in healthcare centres in Africa [11]. A shortened quick SOFA (qSOFA) score (Annex 1) can be applied without any requirement for laboratory tests however qSOFA is not intended to be used as a criterion for diagnosing sepsis, but as a screening tool to identify those with a higher risk of deterioration. Although qSOFA has high levels of specificity for predicting poor outcomes, it is associated with low sensitivity and therefore may not be appropriate for use as a screening tool. By far the most common definition of sepsis currently being used in Africa is the previous systemic inflammatory response syndrome (SIRS) definition (Annex 1) [12]. Defining sepsis using the SIRS criteria leads to the inclusion of an excess of patients with infection or inflammation, and yields low specificity [1, 13-15]. Neither definition of sepsis has been validated in resource poor settings.

**Table 1:** sequential organ failure assessment score reproduced from Singer *et al*. JAMA 2016 [1], sepsis is diagnosed when there is a change in score of 2 or more alongside known or suspected infection

	Score				
System	0	1	2	3	4
Respiration:					
Pa02/Fi02, kPa		≥53.3	<53.3	< 40	<26.7 with respiratory support <13.3 with respiratory support
Coagulation:					
Platelets, x109/L	≥150	<150	<100	<50	<20
Liver:					
Bilirubin, mmol/L	<20	20-32	33-101	102-204	>204
Cardiovasular:					
MAP, mmHgm	≥70	<70			
Catacholamine dose, mg/kg/min			Dopamine <5 or dobutamine	Dopamine 5.1-15 or adrenaline ≤ 0.1 or noradrenaline ≤ 0.1	Dopamine >15 or adrenaline >0.1 or noradrenaline >0.1
Central nervous system:					
GCS	15	13-14	10-12	6-9	<6
Renal:					
Creatinine, mmol/L	<110	110-170	171-299	300-440	>440
Urine output, ml/d				<500	<200

Early warning scores such as the modified early warning score (MEWS) are familiar to clinicians and are increasingly being used as the basis for deciding which patients with infection to treat as sepsis. Such scores, including a newly developed Universal Vital Assessment (UVA) score outperform qSOFA and SIRS at predicting intensive care admission and mortality in sepsis [13, 16-18].

The World Health Organisation (WHO) in 2011 published the Integrated Management of Adult and Adolescent Illness (IMAI) District Clinical Manual (DCM), setting out a clinical definition of severe sepsis/septic shock (Annex 1) which is highly practical in the resource limited setting [19]. It was created by a consensus of international experts recognising the distinct lack of research data and the need for universal guidance for the recognition and management of sepsis, however has yet to be updated and standardised in view of the 3rd International Consensus definitions for sepsis [20].

An evidence based operational definition of sepsis for Africa is urgently required, for use by clinicians, institutions, researchers and policy makers. In practice, clinicians should diagnosis sepsis in the context of a patient with infection and any available clinical or biochemical marker of end organ dysfunction. This should include features such as reduced consciousness and inability to stand and walk independently which has been shown to be associated with higher mortality in Africa [17,21].

### What is the burden of sepsis in Africa?

Through leveraging the Global Burden of Diseases study, Rudd and colleagues estimated that 85% of deaths from sepsis occur in low- and middle-income countries, with the highest burden in Africa [3]. However, there are limited accurate population incidence data from Africa. The problem of data sparsity is exacerbated by the lack of a universal definition of sepsis. Using routine-care data for 217149 patients combined with census data in Blantyre, Malawi, Lewis et al. estimated the incidence of sepsis and severe sepsis (as defined by SIRS criteria) to be 1754-1759 and 295-310 per 100,000 person years respectively between 2013 and 2016 [22]. In a study of 6020 adult surgical admissions in South Africa, 1240 (21%) met criteria for sepsis [23]. An analysis of consecutive admissions to intensive care unit in Rwanda showed that within 24 hours of admission, 42% had a diagnosis of sepsis, 33% severe sepsis, and 21% septic shock [24]. In a survey of 196 consecutive adult medical admissions in Cameroon 33% had sepsis and 21% had septic shock (our unpublished data). Several studies which have enrolled patients with sepsis in a variety of settings have reported high mortality: 7% in Gabon [25], 13% in South Africa [23], 15% in Ethiopia [26], 22-24% in Malawi rising to 28- 50% for severe sepsis [22,27], 23-32% rising to 46% for severe sepsis in seven studies in Uganda [28-33], 40% in Zambia rising to 62% for severe sepsis in two studies in Zambia [10,34] and 64% rising to 83% for septic shock in intensive care in Rwanda [24]. Given the change in definition of sepsis with sepsis now used to refer to patients who were previously described as having severe sepsis, the mortality for sepsis in Africa is estimated to be 30-47% [12].

### How does triage and emergency care affect management of sepsis?

Interventions in sepsis are time critical and delays in treatment worsen outcomes. Even the most well-resourced health systems struggle to deliver the recommended care in sepsis within the recommended timeframes [35]. In low resourced health systems where there are greater barriers to the delivery of timely care to patients, the recognition of sepsis and the triage of patients with poor prognostic features presents a significant challenge. The best validated triage score in low resourced health systems is the South African Triage Score (SATS) using both a list of indicator conditions as well as a Triage Early Warning Score calculated from physiological measurements. SATS has been demonstrated to improve time to intervention in South Africa and other low-resource health systems [36-40]. However SATS has been documented to both over-triage and under-triage patients in several countries in Africa [38,40]. A recent collaborative project has established the UVA scoring system for sepsis validated with data from 6 different countries in Africa (Table 2). A score of 2 to 4 predicted a 17% mortality and a score > 4 predicted a 37% mortality. Furthermore, the UVA score outperformed both qSOFA and MEWS score in sensitivity, specificity and positive predictive value for mortality [13,17]. The uptake, efficiency and safety of triage in the African setting is largely undocumented in the literature, however from the authors collective experience, the triage process is highly variable, non-standardised and should be the focus of a concerted effort to strengthen health systems. We advocate that sepsis education in low resourced health systems is coupled with triage education to encourage clinicians and systems to respond in a timely manner to urgent clinical need regardless of the cause, which in turn will facilitate prompt recognition and management of the septic patient.

**Table 2:** clinical variables and their cut offs in the derivation of the UVA (universal vitals assessment) score reproduced from Moore *et al*. BMJ Global Health 2017 [17]

	Score				
Variable	0	1	2	3	4
Temperature (°C)	≥36		<36		
Heart rate (beats per minute)	<120	≥120			
Respiratory rate (breaths per minute)	<30	≥30			
Systolic blood pressure (mmHg):	≥90	<90			
Oxygen saturations (%)	≥92		<92		
Glasgow Coma Scale	15				<15
HIV infection	No/unknown				

### What do we know about management of sepsis in Africa?

Effective sepsis management requires rapid administration of antibiotics, restoration of normal physiology and tissue perfusion, and the control of the infectious source. Despite the limited evidence for effective interventions in Africa, the same approach is also likely to be of benefit in Africa [41].

### Antibiotics

Administration of broad-spectrum antibiotics within one hour is a universal standard in sepsis care. There is increasing evidence that delay in administration of antibiotics is associated with increased mortality [4,19,42,43]. Several studies in Africa have found no difference in antibiotic administration time among survivors and non-survivors; with 32% receiving antibiotics within 1 hour in Uganda [32], and in Malawi where no difference in antibiotic usage was observed between survivors and non-survivors [27]. In a follow up study in Uganda assessing the impact of protocolised care, the proportion of patients receiving antibiotics within an hour increased from 32% to 67% , and mortality decreased by 13%, however there was no difference between mortality in groups receiving antibiotics within one hour and within six hours [44]. Furthermore the study design was a before and after study with 2 year interval between the two arms so improvement in mortality could have been multifactorial. Nonetheless, antibiotic administration within one hour for the septic patients, especially patients with septic shock should remain a target for sepsis management in any setting. The type of antibiotic prescribed should be tailored to the situation in accordance with known local resistance patterns, particularly as infection with organisms resistant to first line antibiotic therapy are common in Africa and associated with very high mortality [26]. In addition, antimicrobial therapy must be reviewed within 72 hours of prescription in conjunction with microbiological culture results to inform de-escalation of therapy decisions.

### Fluid therapy

The optimal volume of fluid resuscitation in Africa remains controversial. Intravenous fluid should be used to restore hypovolaemia and improve end-organ hypoperfusion, yet published guidance is heterogeneous and may not be readily transferable to African settings [45]. The surviving sepsis campaign recommends using up to 30ml/kg of crystalloid fluid (i.e > 2L in a 70kg adult) as an initial bolus [4]. The WHO IMAI DCM recommends a fluid bolus of 1L followed by a 20ml/kg/h infusion of up to 60ml/kg in septic shock. Compliance with IMAI guideline´s showed no benefit in mortality in a study of 122 patients in Uganda [28]. A small randomised controlled trial of aggressive fluid resuscitation (up to 6L within the first 6 hours) in Zambia was stopped early due to increased mortality from hypoxic respiratory failure [10]. The high profile FEAST trial also found higher mortality in febrile children who received bolus fluid administration in Africa [9]. Effective monitoring of the response to fluid therapy is therefore crucial. Strategies that have proven effective to guide fluid bolus therapy in sepsis include measurement of stroke volume in response to passive straight leg raise (as a marker of fluid responsiveness), and titration of fluid to normalise capillary refill time [46,47]. While ongoing research is required to identify safe and effective fluid resuscitation strategies for patients with sepsis in Africa, the cautious administration of intravenous fluid is reasonable. A practical clinical approach is to use small boluses of fluid coupled with frequent assessment of capillary refill time and fluid status before prescribing further boluses. In addition, fluid therapy should be stopped if there is suspected fluid overload causing respiratory distress.

### Care bundles, monitoring and protocolisation

Early Surviving Sepsis Campaign guidance focused on time specific Early Goal Directed Therapy (EGDT); a series of intensive-care reliant physiological targets aimed at restoring normal physiology and controlling the source of infection. However, three large randomised controlled trails (the ARISE, ProCESS and ProMISe trials) showed no difference in outcomes between those receiving EGDT and routine care [48-51]. Consequently the emphasis of the Surviving Sepsis Campaign guidelines has moved away from EGDT to clinical monitoring of the septic patient. In the African setting where the monitoring required for EGDT (central venous oxygen saturation and pressure) is often not possible, the clinical monitoring of response via blood pressure, urine output, capillary refill time, fluid balance, jugular venous pressure, respiratory rate, oxygen saturations and chest examination to ensure that organ perfusion is adequate and that fluid overload is avoided should be promoted. This recommendation is further supported by a single study of protocolised sepsis care from Uganda demonstrating a reduction in mortality of 13% compared to a baseline observational study in a similar cohort of patients relied on a robust resuscitation protocol with frequent monitoring of treatment to guide fluid therapy and to recognise fluid overload [44]. Another Ugandan study showed an increased mortality among patients who were more frequently monitored, however this is likely to reflect the increased monitoring in critically unwell patients [31]. A simple and easy to remember sepsis bundle such as the sepsis 6 developed and promoted by the UK Sepsis Trust is required for Africa [52]. In the meantime it seems reasonable to adapt the “sepsis 6” bundle, depending on resource availability, whilst research clarifies the most effective interventions for improving outcomes in Africa.

### What investigations should be carried out in patients with suspected sepsis in Africa?

#### Microbiological samples

An integral component of sepsis care in well-resourced health systems is the obtaining of microbiological samples to confirm infection, identify the causative organism and guide antimicrobial de-escalation. Furthermore, from a healthcare system perspective it is important to use knowledge of local patterns of antimicrobial resistance to design empirical antibiotic regimens to ensure good antimicrobial coverage against the likely causative organisms. Antimicrobial resistance is a significant problem in Africa and it is estimated that 10% of patients in Africa have a healthcare associated infection [53]. A systematic review of blood stream infections in Africa revealed that from 15166 adults from 22 studies who had prospectively taken blood cultures, 13.5% had positive blood cultures with Salmonella enterica, Streptococcus pneumoniae, Staphylococcus aureus and Escherichia coli the most prevalent bacteria [54]. However these studies are limited to only twelve countries in Africa, and predominantly from academic centres. The effective integration of microbiological services to support the clinical and health system management of sepsis is a significant challenge in many low resource settings and further strengthening of health systems is desperately required to achieve this in Africa.

#### Lactate measurement

The use of lactate as both a marker of end organ hypoperfusion and as a prognostic marker in sepsis has been well documented [55,56]. Lactate clearance is associated with better outcomes, thus lactate clearance can be used as a target for fluid resuscitation [57-59]. Lactate measurement has been shown to predict mortality in an observational study of patients with sepsis in Uganda [60]. One other study has assessed lactate clearance as a marker of response to resuscitation in Africa. In this study of 202 patients in Uganda, patients who cleared lactate by 10% at six hours had received significantly more IV fluids, but lactate clearance did not correlate with improved outcomes [29]. Although lactate measurement is simple and can be performed at the bedside as a point of care test, it may not be available in many settings in Africa. In Chile (middle-income country) a trial comparing fluid resuscitation guided by capillary refill assessment versus lactate measurement showed no difference in 28-day mortality [47]. Pending further research, it is reasonable to use lactate measurement to assist in the recognition and management of sepsis in Africa where available, and use capillary refill time assessment where it is not available.

#### Malaria and TB diagnostics

The differential diagnosis of the acute febrile illness in Africa must include consideration of malaria and tuberculosis. In malaria endemic regions, bacterial sepsis is often clinically mistaken for malaria and can cause delays to appropriate management with antimicrobials [61]. Mycobacterium tuberculosis (MTB) is a common cause of sepsis in the African setting, particularly in regions with high HIV prevalence [62,63]. One study in Uganda identified MTB bacteraemia in nearly one in four HIV positive patients with severe sepsis [64]. It is therefore crucial that sepsis pathways in malaria endemic regions incorporate malaria rapid diagnostic testing to avoid the misdiagnosis of bacterial sepsis as malaria. MTB bacteraemia is more challenging to diagnose, and must be considered in HIV prevalent regions as a potential cause of sepsis. Measurement of urinary mycobacterial lipoarabinomannan (LAM) is recommended in seriously ill patients with a CD4 count of < 200 cells/μL and has been demonstrated to improve survival in patients with a CD4 count of < 100 cells/μL [65]. Another strategy for tuberculosis diagnosis using bedside ultrasound (focused assessment with sonography for TB in HIV (FASH)) may assist in TB diagnosis but has not been evaluated in controlled trials [66]. There is a case for encouraging clinicians in these settings to have a low threshold for empirical anti-tuberculous therapy in severe sepsis in HIV positive patients, however this must be weighed up against the capacity of healthcare systems to manage the increased case load that could result from this strategy.

### What is the role of intensive care in Africa?

Sepsis management in well-resourced health systems is reliant on intensive care level staffing, monitoring and organ support. The bulk of research and much of the guidance for managing and monitoring in sepsis relies on intensive care facilities. However, these facilities have limited availability in the vast majority of African settings [67]. Consequently, critically ill patients with sepsis are frequently managed on general in-patient wards. The variability in access to respiratory and ventilatory support, blood pressure support with vasopressors and ionotropes and renal replacement therapy remains a particular challenge and the improvement in health systems organisation and funding required for universal intensive care access is unlikely to change in the near future. The WHO IMAI DCM recommends the use of blood pressure support with dopamine or adrenaline (interventions which are largely limited to intensive care environments in well-resourced systems). There is limited evidence to support this use, however in the absence of evidence it is reasonable to attempt to use these agents to reverse refractory hypotension and to attempt to avoid renal failure requiring renal replacement therapy which is not readily available. The absence of high-quality and accessible intensive care facilities in many parts of Africa highlights the importance of focusing on early recognition and management of sepsis outside critical care.

### What are the barriers to high-quality sepsis care in Africa?

Even in the best resourced health systems, implementing the surviving sepsis guidance presents a significant challenge. In Africa these barriers are greater where access to healthcare, hospital resources and staffing are more limited and the burden of infection is higher. Nonetheless, the interventions which are known to have an impact on outcome in sepsis, namely prompt recognition and timely delivery of antibiotics, oxygen, fluid challenges alongside monitoring response to treatment can be implemented in the majority of low resource settings in Africa. It is a considerable challenge to describe institutional practice and, where necessary, to alter institutional behaviour in order to deliver the best treatment to the right patients at the right time [68]. There are encouraging precedents in the literature, where quality improvement projects in Africa have demonstrated improvements in knowledge and behaviours of healthcare professionals in relation to sepsis management [69-71].

### The role of leadership and need for strategic planning

Improving sepsis in Africa requires leadership and organisation of efforts at a local, national and continental level. A group of 60 experts across Africa have recently created the African Sepsis Alliance in order to achieve this. The alliance is supported by the Global Sepsis Alliance and currently involves more than 15 African countries. The vision is to transform sepsis care in Africa by bringing about large-scale improvements in sepsis awareness, education, care, research and funding in Africa. The specific objectives of the alliance are to:

1) Promote sepsis prevention, including vaccination against bacterial infections and interventions aimed at infection prevention/control and reducing healthcare associated infections. 2) Promote education amongst healthcare workers, members of the public and other stakeholders. 3) Promote coordinated research and development in Africa and set priorities such as estimating the burden of disease, causes, prognosis and effective interventions. 4) Promote quality improvement with a focus on pre-hospital care and care in out-of-critical care environments (emergency departments and wards). This will involve highlighting and promoting good practice as well as quality improvement training, leadership and development of tools to support sepsis improvement, similar to initiatives in the western world. 5) Lobby and ensure sepsis becomes a recognised priority in health systems in Africa. 6) Fundraise and generate large amounts of resources to support education, quality improvement and research into sepsis in Africa.

These objectives fall in line with the WHO sepsis resolution passed in May 2017 and the first meeting of the alliance took place in Uganda in October 2017 during the African Federation of Critical Care Nurses Association Conference. In 2019, the NIHR funded the African Research Collaboration on Sepsis (ARCS) which is led by the Liverpool School of Tropical Medicine. This research collaboration has sites across 10 countries in Africa and involves a number of important studies, including a short incidence study of sepsis in adults and a Delphi study to determine quality indicators for critical care and sepsis in low- and middle-income countries. The outcomes of these studies are eagerly awaited. However, there is a need for large-scale quality improvement programs in most African countries, and this can be achieved by development of national action plans for sepsis [72]. These plans should be developed by national Ministries of Health working with stakeholders such as the Global Sepsis Alliance, African Sepsis Alliance, CDC Africa, African Union and the WHO. In so doing, African countries will align with statements in the 2017 WHO Sepsis Declaration. In addition, fortification of African health systems will benefit not only patients with sepsis in the short term but the whole population in the long term.

### Summary

Advances in sepsis management in well-resourced healthcare systems, have not been effectively transferred to the African setting where the burden is greatest. We recommend that every African nation develops a national action plan for sepsis in order to build sepsis research capacity, strengthen healthcare systems and improve the outcomes for patients with sepsis.

## Annex

**Annex 1**: box 1, box 2, box 3
